# Effects of converting *Eucalyptus* plantations to six native tree species on microbial nutrient limitation in subtropical plantation soils

**DOI:** 10.3389/fmicb.2026.1770355

**Published:** 2026-03-16

**Authors:** Yongmei Xiong, Seping Dai, Yu Su, Yanqiong Li, Jianmin Xu

**Affiliations:** 1 Research Institute of Tropical Forestry, Chinese Academy of Forestry, Guangzhou, China; 2 Nanjing Forestry University, Nanjing, China; 3 Guangzhou Institute of Forestry and Landscape Architecture, Guangzhou, China; 4 Guangzhou Collaborative Innovation Center on Science-Tech of Ecology and Landscape, Guangzhou, China

**Keywords:** *Eucalyptus* plantation, forest conversion, microbial nutrient limitation, native tree species, soil depth, soil enzymes

## Abstract

The selection of tree species is critical for restoring ecosystem functions in degraded forests, yet the impacts of native species reintroduction on belowground microbial processes remain poorly understood, particularly across soil profiles. Here, we measured the potential activities of two C-acquiring enzymes (*β*-1,4-glucosidase and *β*-D-cellobiosidase), two N-acquiring enzymes (*β*-N-acetylglucosaminidase and leucine aminopeptidase), and one organic-P-acquiring enzyme (alkaline phosphatase). Using variance analysis, correlation analysis, redundancy analysis (RDA), random forest analysis (RFA), we quantify and compared the variations in microbial resource limitations in 0–10 cm surface and 20–30 cm subsurface soils following converting *Eucalyptus* to six native tree species plantations. Results showed that after conversion to native tree species, surface C-acquiring enzyme activity decreased whereas N- and P-acquiring enzymes showed no significant differences across most plantations; in contrast, subsurface soils exhibited a consistent increase in C-, N-, and P-acquiring enzyme activities. RDA showed that C-, N-, and P-enzymes were mainly influenced by soil microbial biomass and N content. Microbial C limitation was significantly alleviated but P limitation intensified in surface soils, while microbial C and P limitation in subsurface soils showed no significant change across most plantations following conversion. RFA showed that surface C limitation was mainly influenced by microbial biomass stoichiometric ratios, while subsurface C limitation was mainly regulated by NO_3_^−^ and bulk density. Surface P limitation was primarily driven by soil N content (NH₄^+^, TN and C:N), whereas subsurface P limitation showed no significant driver. These findings highlight introducing native tree species restructures microbial nutrient limitation patterns and functions, underscoring the potential of native species to improve belowground ecological processes in plantation ecosystems and providing mechanistic insights for tree species selection.

## Introduction

The process of forest ecological succession not only profoundly reshapes the structure and composition of forest communities but also regulates soil microbial metabolism and energy flow patterns by influencing the quantity and quality of soil organic matter, soil physicochemical properties, root exudates, and litter inputs (Tian et al., 2015; Deng et al., 2016; Zhao et al., 2018; Sun et al., 2025). These shifts largely determine the role and efficiency of soil microbes in the cycling of carbon (C) and key nutrients such as nitrogen (N) and phosphorous (P), thereby further shaping soil structure and ecosystem functions (Sinsabaugh and Shah, 2012; Moorhead et al., 2016). However, a consensus is still lacking regarding the specific effects of afforestation and forest management on plant community succession, and how these effects drive changes in substrate availability, enzyme activity, microbial composition, and nutrient resources (Li et al., 2014; Naveed et al., 2016). Understanding microbial metabolism and its limitation is pivotal for global challenges and harnessing microbial capabilities for sustainable development due to the combined influences of tree species selection, afforestation patterns, climate conditions, and soil types.

Soil microorganisms are the primary driver of ecosystem processes and serve as the foundation of detrital food webs, regulating soil nutrient cycling and maintaining the stoichiometric balance among C, N, and P in terrestrial ecosystems (Waring et al., 2013; Peng and Wang, 2016; Deng et al., 2019). Variations in soil microbial metabolism currently represent one of the greatest uncertainties in understanding soil nutrient cycles and predicting terrestrial C sinks (Liang et al., 2017; Malik et al., 2020). Soil extracellular enzymatic activity (EEA) reflects the biogeochemical balance between nutrient requirements of microbial assemblages and nutrient availability of the environment (Moorhead et al., 2013; Rosinger et al., 2019). Soil EEA stoichiomertry reflects how the microbial community invests in energy and nutrient acquisition under *in situ* conditions as it copes with nutrient limitation (Tapia–Torres et al., 2015). Bowles et al. (2014) reported soil microbes regulate extracellular enzyme production to acquire limiting nutrients, thus changes in enzyme activities may reflect patterns of microbial nutrient limitations and hence nutrient availability. Microbial enzymes activities are widely used to reveal microbial metabolic limitations related to C, N, and P (Moorhead et al., 2016; Cui et al., 2022). To illuminate the characteristics of microbial metabolism, Moorhead et al. (2016) proposed calculating the “lengths” and “angles” of vectors in a plot of proportional activities of enzyme C:N vs. C:P acquisition to quantify the relative investments in C versus nutrient acquisition (vector lengths) or P versus N acquisition (vector angles). However, the resource utilization and nutrient limitation of soil microorganisms mediated by EES was varied in different ecosystems due to climate, vegetation and soil types (Peng and Wang, 2016; Wang et al., 2023; Schaap et al., 2023; Zhang et al., 2020).

Forest vegetation succession, soil physicochemical properties, nutrient stoichiometry, and climatic conditions jointly regulate soil EEA and their functions. For example, vegetation succession and types directly influence the soil environment by altering aboveground and belowground biomass, root exudation, and litter inputs, which in turn regulate the structure and function of soil microbial communities (Xu et al., 2015, 2021; Sun et al., 2025; Sveen et al., 2025). Meanwhile, soil moisture directly influence soil metabolic processes and EEA (Geisseler et al., 2011; Bi et al., 2022; Cui et al., 2020). Soil nutrients and soil texture significantly affect soil microbial metabolism through directly and indirectly (Snajdr et al., 2008; Zhao et al., 2018). In fact, soil C-N-P stoichiometry modulates EEA by influencing soil biochemical properties and regulating enzyme secretion (Moorhead et al., 2016; Sinsabaugh et al., 2009; Bing et al., 2016). In addition, different tree species alter soil microenvironment and microbial community structure through variations in root traits, litter quality and decomposition rates, as well as root exudates, thereby influencing the types and activity levels of soil enzymes (Prescott, 2010; Prescott and Grayston, 2013; Ushio et al., 2010). Therefore, it is necessary to systematically investigate how afforestation with different tree species alters soil properties and the limiting factors and adaptive mechanisms of soil microbial metabolism through plant–soil interactions, which is crucial for understanding forest management and silvicultural practices.

*Eucalyptus*, is widely recognized as a fast-growing plantation species in tropical and subtropical regions, valued for its rapid growth, strong adaptability, and considerable economic potential. However, as *Eucalyptus* plantations have expanded rapidly, the prevalence of monoculture systems and long-term uniform management practices, together with resulting soil nutrient depletion and water use pressures, have aroused various ecological and environmental challenges. Transforming *Eucalyptus* plantations is regarded as an important measure to mitigate these issues. In this study, we clear-cut *Eucalyptus* monocultures and replanted six native tree species to investigate how different native tree afforestation types influence soil physicochemical properties, nutrient acquisition, and microbial characteristics. We aimed (i) to assess how *Eucalyptus* conversion affects soil ecoenzymatic activities and microbial nutrient limitations in surface and subsurface soils, and (ii) to explore how soil environmental factors shape these parameters in subtropical plantations. We hypothesized that replacing *Eucalyptus* with native tree species would enhance soil nutrient status and microbial enzyme activities, alleviate microbial nutrient limitations, and that these effects would be more pronounced in surface soils than in subsurface soils due to plant–soil interactions.

## Materials and methods

### Study area and soil sampling

The study was conducted at Zengcheng Taizikeng Forest Park (23°05′ ~ 23°37′N, 113°29′ ~ 114°00′E) in Guangzhou city, Guangdong province, China. The region has a subtropical monsoon climate, characterized by abundant thermal resources and rainfall, with an average annual temperature of 22.2 °C and an average annual precipitation of approximately 1869 mm. A silvicultural experiment aimed at enhancing forest quality was implemented in this park, which involved clear-cutting existing *Eucalyptus* stands and replanting the areas with native tree species in 2002. To compare forest conversion strategies, a control plot of monoculture *Eucalyptus* was maintained alongside six experimental plots, all at a consistent planting density. In the experimental plots, the eucalyptus was replaced with monocultures of the following native species: *Michelia macclurei* (*M. macclurei*), *Rhodoleia championii* (*R. championii*), *Erythrophleum fordii* (*E. fordii*), *Mytilaria laosensis* (*M. laosensis*), *Castanopsis hystrix* (*C. hystrix*), and *Michelia chapensis* (*M. chapensis*). In each monoculture forest, three 20 × 20 m plots were established, yielding a total of 21 plots. In August 2024, soil samples were collected along an S-shaped pattern. Within each plot, two soil layers were sampled: surface (0–10 cm) and subsurface (20–30 cm), resulting in total of a 42 soil samples.

### Soil physiochemical analysis

Soil organic carbon (SOC) was determined colorimetrically using dichromate oxidation when boiling with a mixture of potassium dichromate and sulfuric acid. Soil total N (TN) and total P (TP) were determined using a continuous flow analyzer following wet digestion: TN with HClO_4_–H_2_SO_4_, and TP with H_2_SO_4_ in the presence of a K_2_SO_4_:CuSO_4_·5H_2_O catalyst (10:1 w/w) (Zhang et al., 2019a,b). Based on these measurements, the stoichiometric ratios of SOC to TN (C:N), SOC to TP (C:P), and TN to TP (N:P) were calculated. Soil available phosphorus (AVP) was determined using the molybdenum blue colorimetric method. Soil pH (1:2.5 soil/water ratio) was measured using a pH meter (FE20K, Mettler-Toledo). Soil ammonium nitrogen (NH_4_^+^) and nitrate nitrogen (NO_3_^−^) concentrations were analyzed using an auto-analyzer (FIAstar 5,000, FOSS). Soil bulk density (BD) and soil water content (SWC) were determined using the core ring knife method. Soil microbial biomass carbon (MBC), nitrogen (MBN), and phosphorus (MBP) were measured using the chloroform fumigation-extraction method (Brookes, 1995). The ratio of MBC to MBN (MB_C:N_), MBC to MBP (MB_C:P_), MBN to MBP (MB_N:P_) were then calculated.

### Assays of extracellular enzyme activities

Focusing on five enzymes associated with soil C, N and P cycling, including two C-acquiring enzymes: *β*-1,4-glucosidase (BG) and *β*-D-cellobiosidase (CBH); two N-acquiring enzymes: *β*-N-acetyl glucosaminidase (NAG) and leucine aminopeptidase (LAP) and one P-acquiring enzyme: acid/alkaline phosphatase (AP). Soil extracellular enzyme activities were measured using fluorometric assays according the method of Saiya–Cork et al. (2002). The five enzymes have also been widely used as indicators of C-, N-, and P-acquiring enzymes in previous studies on extracellular enzyme activity stoichiometry at both global and regional scales.

### Quantification of microbial metabolic limitation

Microbial metabolic limitation was quantified by calculating vector length and angle of enzymatic activity from untransformed proportional activities, e.g., (BG + CBH)/(BG + CBH + NAG+LAP). The vector length, representing microbial C limitation, was computed as Length=SQRT (*x*^2^ + *y*^2^), where *x* denotes the relative activity of C- versus P-acquiring enzymes, and *y* represents the relative activity of C- versus N-acquiring enzymes (Moorhead et al., 2013, 2016). The vector angle, reflecting microbial N or P limitation, was calculated as the arctangent of the line extending from the origin to coordinate point (*x*, *y*), i.e., Angle(°)=DEGREES(ATAN2(x,y)) . Microbial C limitation increases with vector length. Vector angles >45° signify microbial P limitation, and vector angles <45° denote microbial N limitation. As the vector angle increases, microbial P limitation becomes more pronounced, while microbial N limitation diminishes.

### Statistical analysis

First, a one-way analysis of variance (ANOVA) followed by Tukey’s test was applied to evaluate the effects of different tree species treatments on soil physicochemical properties, microbial biomass and microbial metabolic limitation after the clear-cuting of *Eucalyptus* plantations. All date are showed as the mean ± standard error (SE). Meanwhile, we tested the effects of tree species, soil depth and their interaction on soil ecoenzymatic activities (BG + CBG, NAG+LAP, and AP) and vector characteristics (vector length and vector angle) using two-way ANOVA. Second, Pearson’s correlation analysis was employed to examine the associations among soil physicochemical properties, microbial biomass and microbial functional characteristics. A correlation heatmap was created using the “corrplot” package to visualize the Pearson’s correlation coefficients (Wei et al., 2017). Third, redundancy analysis (RDA) was carried out to evaluate changes of enzyme activities relation to soil environmental properties. The RDA was performed with the CANOCO 5.0 software package (Šmilauer and Lepš, 2014). Finally, the key environmental factors influencing microbial limitation were determined through a classification random-forest algorithm analysis (RFA). Because many soil variables with complex interrelationships were included, negative %IncMSE values may occur during permutation due to random disturbance and collinearity, and such variables generally contribute little to model prediction. To ensure model robustness, we tested different numbers of trees and compared the consistency of variable importance rankings. Variables were then ranked according to %IncMSE, and only the top five predictors were retained to construct the final models to reduce noise and potential overfitting and to highlight the main controlling factors. The analyses were implemented using the “randomForest” and “rfPermute” packages (Breiman et al., 2018). The significance of the predictors was estimated from their percentage effect on the mean squared error. All statistical analyses and data visualizations were performed using R software version 4.3.6 (R Core Team, 2019).

## Results

### Soil properties and microbial biomass among different tree species plantations

At both the 0–10 cm surface and 20–30 cm subsurface soils, not all soil parameters consistently exhibited higher or lower values in native plantations compared with *Eucalyptus* plantations. In general, native plantations tend to exhibit higher SWC, C, P, C:N, MBP, and NO_3_^−^ levels than *Eucalyptus* plantations, whereas their C:P and N:P ratios are generally lower. In contrast, other properties, such as pH, BD, AVP, NH_4_^+^, microbial stoichiometric characteristics, varied considerably among different plantation types. At the 0–10 cm surface soils, *M. macclurei* had the highest C, N, C:P, NO_3_^−^ and MB_N:P_ contents; *R. championii* showed the highest pH, AVP, MB_C:N_ and MB_C:P_; whereas *C. hystrix* exhibited the highest SWC, NH_4_^+^ MBC, MBN, and MBP contents. At the 20–30 cm subsurface soils, *M. maccl*urei exhibited the highest C, MBC, and MBP contents; *R. championii* showed the highest N, P, AVP, MB_C:N_ MB_C:P_ and MB_N:P_ contents; while *M. chapensis* had the highest C:P, N:P, NH_4_^+^ and NO_3_^−^ contents (Supplementary Tables S1, S2).

### Effect of different tree species plantations on soil extracellular enzyme activities

**Figure 1:**
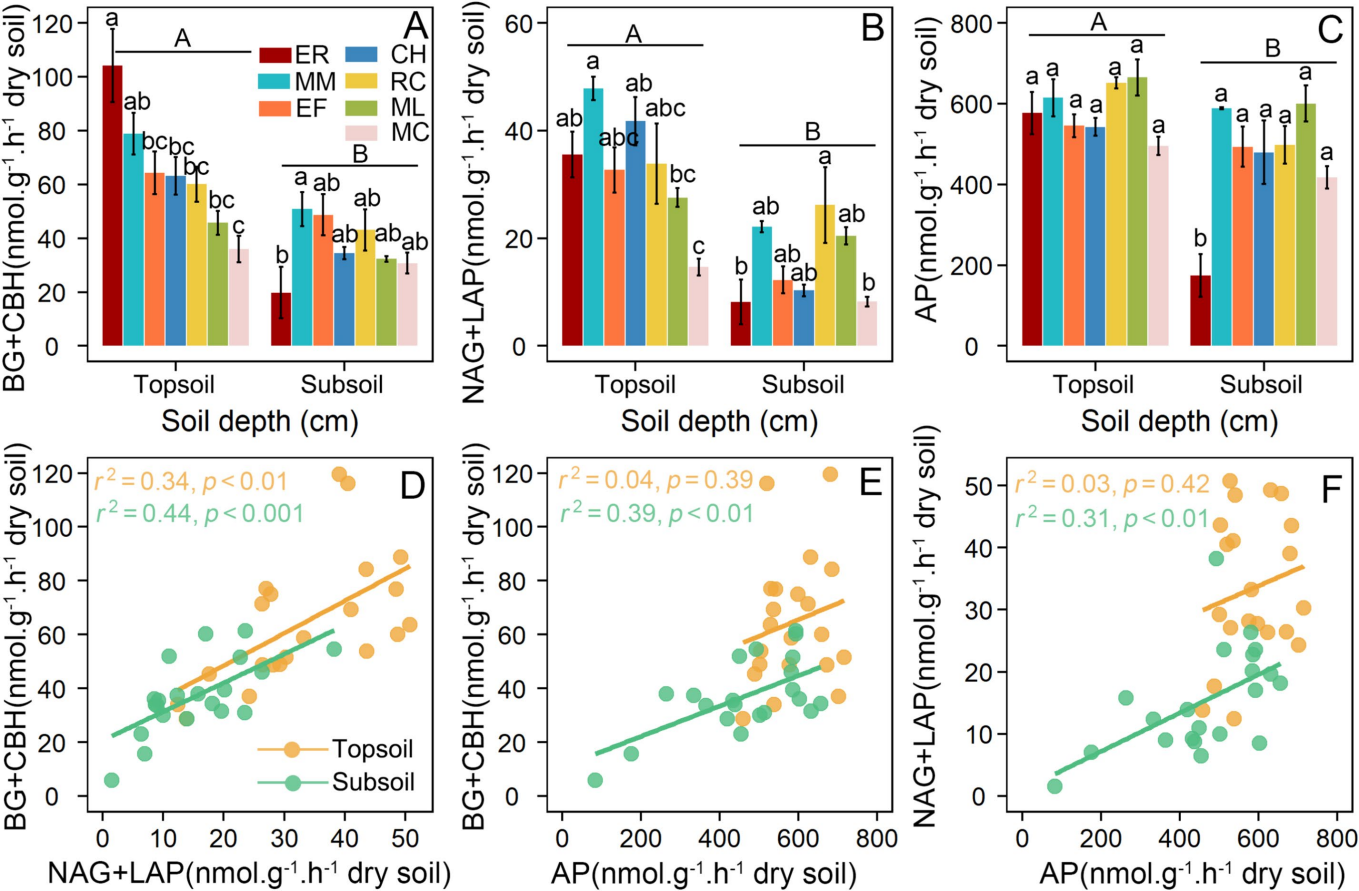
Variations **(A–C)** and correlations **(D–F)** of extracellular enzyme activities among different tree species plantations after the clear-cutting of *Eucalyptus* plantations. Different letters above the bars indicate significant differences between treatments (*p* < 0.05). Lowercase letters indicate differences within the same soil layer, while uppercase letters indicate differences between surface and subsurface soils. ER, *Eucalyptus robusta*; MM*, Michelia macclurei*; EF, *Erythrophleum fordii*; CH, *Castanopsis hystrix*; RC, *Rhodoleia championii*; ML, *Mytilaria laosensis*; MC, *Michelia chapensis*; BG, *β*-1,4-glucosidase; CBH, *β*-D-cellobiosidase; NAG, *β*-N-acetyl glucosaminidase; LAP, leucine aminopeptidase; AP, acid phosphatase.

Two-way ANOVA revealed that soil depth demonstrated statistically stronger effects than different tree species and their interaction, as evidenced by consistently higher *F*-values and *p*-values for C-, N- and P-acquiring enzyme activities (Table 1). At 0–10 cm surface soils, soil C-acquiring enzyme activities in *Eucalyptus* plantations were consistently significantly higher than those in six native tree species plantations (Figure 1A). N-acquiring enzyme activities in *Eucalyptus* plantations were significantly higher than those in *M. laosensis* and *M. chapensis* plantations, but showed no significant differences compared with the other four native tree species plantations in surface soils (Figure 1B). P-acquiring enzyme activities did not differ significantly between the *Eucalyptus* and six native tree species plantations in surface soils (Figure 1C). However, at 20–30 cm subsurface soils, C-, N-, and P-acquiring enzyme activities in *Eucalyptus* plantations were consistently significantly lower than those in the six native tree species plantations (Figures 1A–C). Meanwhile, strong positive correlations were observed between C- and N-acquiring enzyme activities in the surface and subsurface layers (*r*^2^ > 0.34, *p* < 0.01; Figure 1D). Although no significant positive correlations were found between C- and P-acquiring or N- and P-acquiring enzyme activities in surface soils (*r*^2^ > 0.03, *p* < 0.05), significant positive correlations were detected in subsurface soils (*r*^2^ > 0.31, *p* < 0.01; Figures 1E,F).

**Table 1:** Two-way ANOVA results showing the effects of tree species, soil depth and their interaction on soil extracellular enzyme activities and vector characteristics.

Variable	BG + CBG	NAG+LAP	AP	Angle	Length
Species	5.25***	7.56***	8.78***	5.70**	8.91***
Depth	51.90***	79.45***	28.09***	26.56***	17.84***
Species × Depth	6.84***	4.04**	4.77**	2.14^ns^	4.51**

Values are F-statistics from two-way ANOVA. BG, *β*-1,4-glucosidase; CBH, *β*-D-cellobiosidase; NAG, *β*-N-acetyl glucosaminidase; LAP, leucine aminopeptidase; AP, acid phosphatase. Significance levels are indicated as *** *p* < 0.001; ** *p* < 0.01; * *p* < 0.05; ns *p* > 0.05.

### Influence of different tree species plantations on microbial metabolic limitation

**Figure 2:**
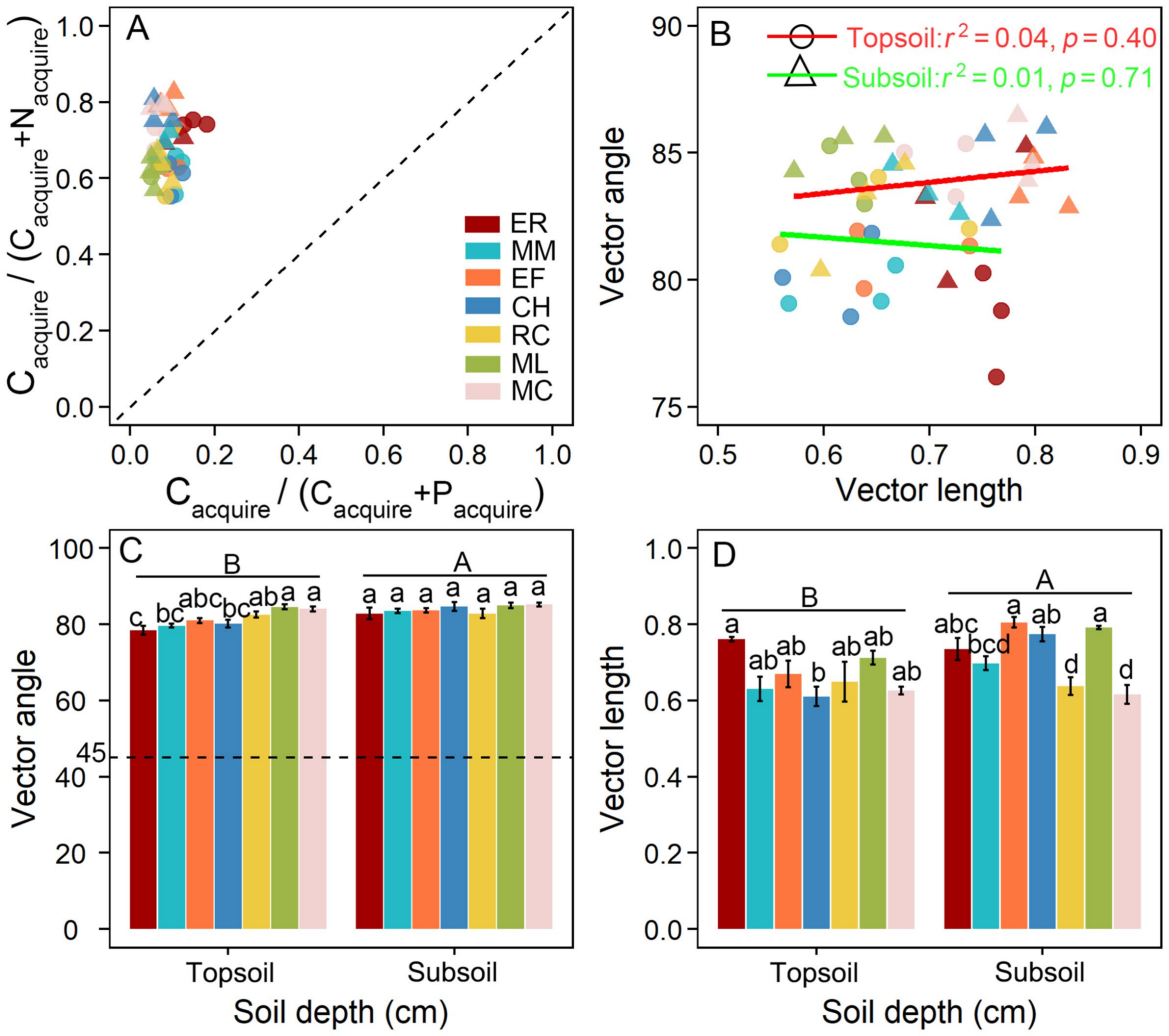
Extracellular enzyme stoichiometry of the relative proportions of C to N acquisition versus C to P acquisition **(A)** and their relationship **(B)**, the variation of vector angle and length **(C,D)** under different tree species plantations after the clear-cutting of *E. robusta* plantations. Different letters above the bars indicate significant differences between treatments (*p* < 0.05). Lowercase letters indicate differences within the same soil layer, while uppercase letters indicate differences between surface and subsurface soils. ER*, Eucalyptus robusta*; MM*, Michelia macclurei*; EF, *Erythrophleum fordii*; CH, *Castanopsis hystrix*; RC, *Rhodoleia championii*; ML, *Mytilaria laosensis*; MC, *Michelia chapensis*.

The characteristics of ecoenzymatic stoichiometry exhibited distinct patterns under different tree species plantations after the clear-cutting of *E. robusta* plantations. All data points positioned above the 1:1 line showed a pronounced P limitation in the microbial community (Figure 2A). The relative C and P limitation of microbes was quantified by calculating the vector lengths and angles. Linear-regression analysis revealed no significant correlation between C and P limitation in either the 0–10 cm surface (*r*^2^ = 0.01, *p* = 0.71) or the 20–30 cm subsurface soils (*r*^2^ = 0.04, *p* = 0.40; Figure 2B).

Two-way ANOVA revealed that different tree species, soil depth and their interaction had significant effects on vector length and vector angle. Soil depth had a more pronounced effect on enzymatic vector characteristics, followed by tree species and their interaction effects (Table 1). The vector angles of all tree species plantations ranged from 76.17° to 85.36° in surface soils and from 79.91° to 86.47° in subsurface soils, all exceeding 45°, indicating pronounced P limitation. Compared with the six native tree species plantations, P limitation in *Eucalyptus* plantations was generally lower, with significant differences particularly compared with *R. championii*, *M. laosensis* and *M. chapensis* in surface soils, whereas no significant differences were observed in subsurface soils (Figure 2C). However, C limitation in *Eucalyptus* plantations was slightly higher than that of six native tree species plantations, but the difference were not statistically significant in surface soils, except for *C. hystrix*. Specifically, in subsurface soils, C limitation in *Eucalyptus* plantations was significantly higher than that in *R. championii* and *M. chapensis* plantations, but did not significantly differ from that in other native tree species plantations (Figure 2D).

### Relationships among soil extracellular enzyme activities, vector characteristics and multiple environmental factors

**Figure 3:**
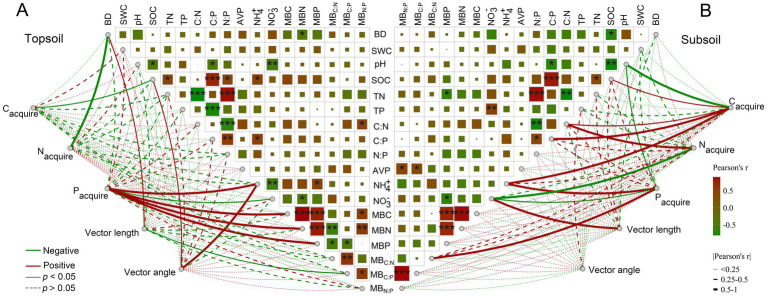
Correlations between extracellular enzyme activities and microbial vector characteristics, and individual environmental factors. BD, bulk density; SWC, soil water content; SOC, soil organic carbon; TN, total nitrogen; TP, total phosphorus; C:N, SOC: TN ratio; C:P, SOC: TP ratio; N:P, TN: TP ratio; AVP, available phosphorus; NH_4_^+^; ammonium nitrogen; NO_3_^−^, nitrate nitrogen; MBC, microbial biomass carbon; MBN, microbial biomass nitrogen; MBP, microbial biomass phosphorus; MB_C:N_, MBC: MBN ratio; MB_C:P_, MBC: MBP ratio; MB_N:P_, MBN: MBP ratio.

At the 0–10 cm surface soils, linear regression analysis showed that SOC was significantly negatively correlated with C-acquiring enzyme activities, and BD was significantly negatively correlated with N-acquiring enzyme activities (both *p* < 0.05). Soil NH_4_^+^, MBC, MBN and MBP were significantly positively correlated with P-acquiring enzyme activities, whereas NO_3_^−^ showed a significant negative correlation (all *p* < 0.05). Additionally, soil BD was significant positively correlated with vector length, whereas MB_C:P_ was significantly negatively correlated (all *p* < 0.05). SOC and NH_4_^+^ were significantly positively related to vector angle (*p* < 0.05; Figure 3A).

At the 20–30 cm subsurface soils, SOC, C:N, C:P and NH_4_^+^ were significantly positively correlated with C-acquiring enzyme activities, whereas pH showed a significant negative correlation (all *p* < 0.05). Soil C:N was significantly positively correlated, whereas NO_3_^−^ was significantly negatively correlated, with N-acquiring enzyme activities (all *p* < 0.05). Soil NH_4_^+^ and MB_C:N_ were significantly positively correlated, whereas pH and NO_3_^−^ were significantly negatively correlated, with P-acquiring enzyme activities (all *p* < 0.05). None of the indices were significantly related to vector angle (*p* > 0.05), whereas NO_3_^−^ was significantly negatively related to vector length (*p* < 0.05; Figure 3B).

### Key factors regulating extracellular enzyme activities and microbial metabolic limitation

**Figure 4:**
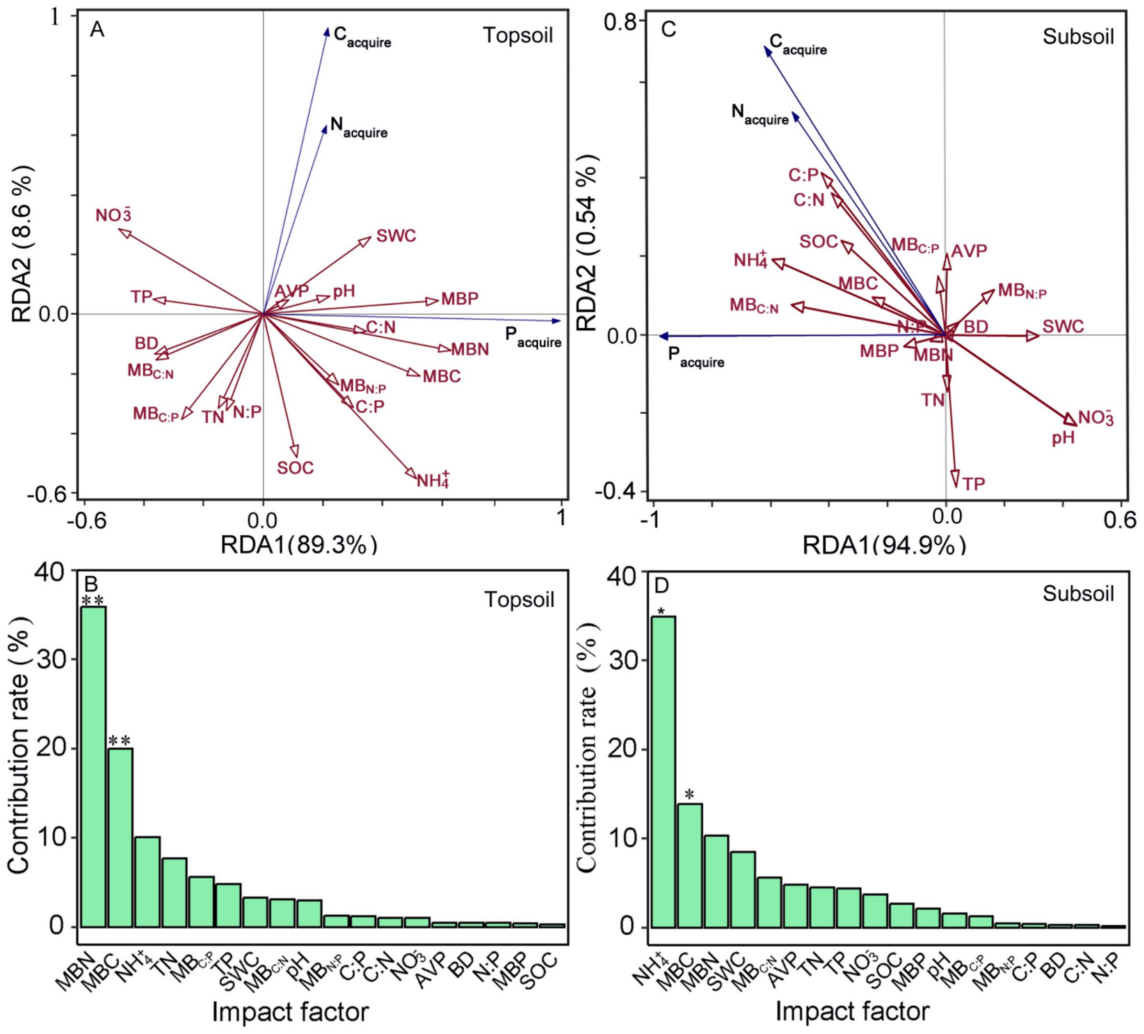
Redundancy analysis conducted to evaluate the relationship between enzyme activities and environmental factors **(A–D)**. BD, bulk density; SWC, soil water content; SOC, soil organic carbon; TN, total nitrogen; TP, total phosphorus; C:N, SOC: TN ratio; C:P, SOC: TP ratio; N:P, TN: TP ratio; AVP, available phosphorus; NH_4_^+^; ammonium nitrogen; NO_3_^−^, nitrate nitrogen; MBC, microbial biomass carbon; MBN, microbial biomass nitrogen; MBP, microbial biomass phosphorus; MB_C:N_, MBC: MBN ratio; MB_C:P_, MBC: MBP ratio; MB_N:P_, MBN: MBP ratio.

The first and second RDA axes explained 89.30 and 8.60% of the variance of soil enzyme-environmental factors at the 0–10 cm surface soils, respectively (Figure 4A). Soil MBN and MBC had significant influences on soil C-, N-, and P-acquiring enzyme activities (Figure 4B). At the 20–30 cm subsurface soils, the first two RDA axes explained 94.90 and 0.54% of the variance, respectively (Figure 4C). Soil NH_4_^+^ and MBC significantly affected soil C-, N-, and P-acquiring enzyme activities (Figure 4D).

**Figure 5:**
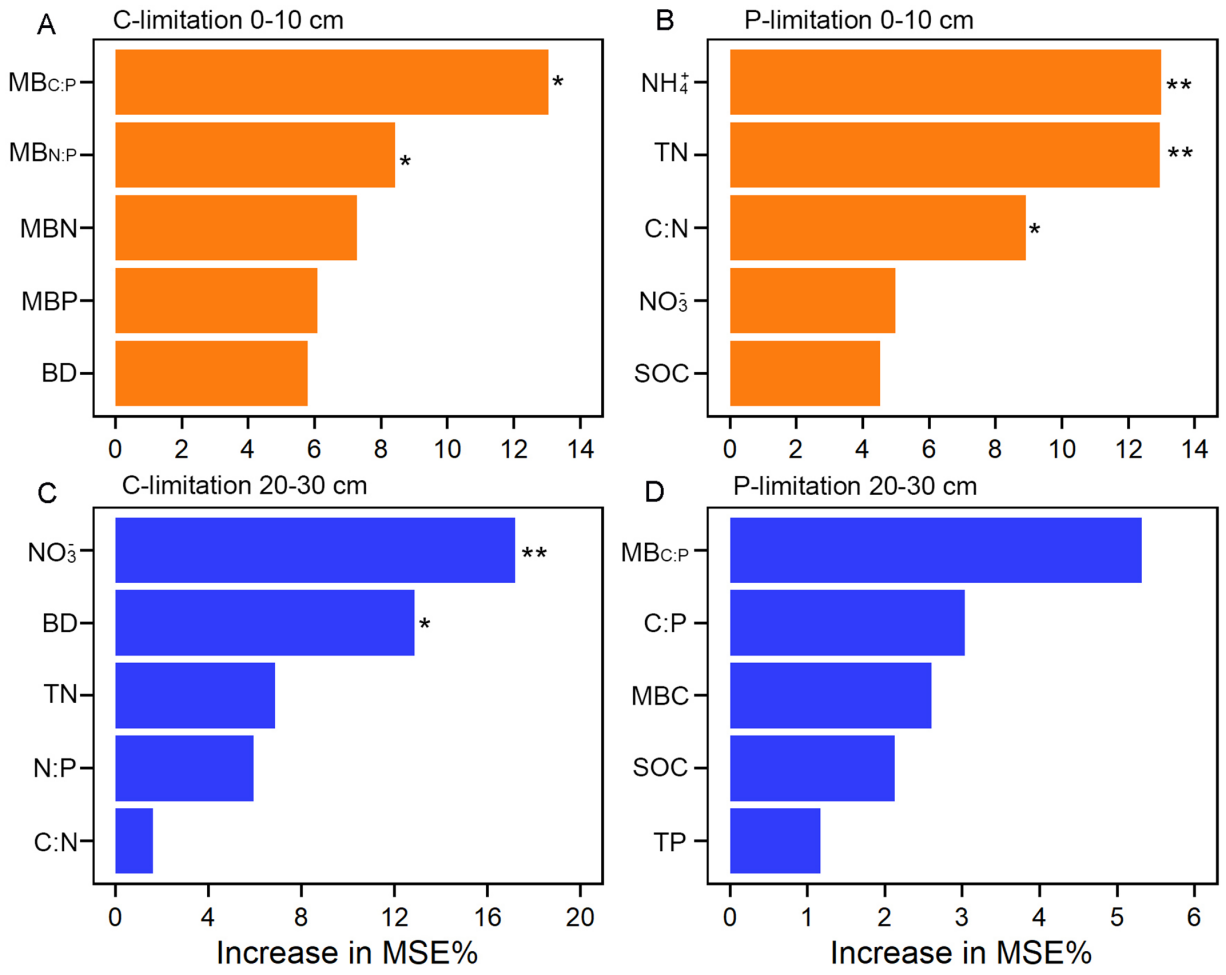
Random-forest models identifying the contributions of each predictor variable in explaining C **(A,C)** and P limitation **(B,D)**. ***, *p* < 0.001; **, *p* < 0.01; *, *p* < 0.05. BD, bulk density; SOC, soil organic carbon; TN, total nitrogen; TP, total phosphorus; C:N, SOC: TN ratio; C:P, SOC: TP ratio; N:P, TN: TP ratio; NH_4_^+^; ammonium nitrogen; NO_3_^−^, nitrate nitrogen; MBC, microbial biomass carbon; MBN, microbial biomass nitrogen; MBP, microbial biomass phosphorus; MB_C:N_, MBC: MBN ratio; MB_C:P_, MBC: MBP ratio; MB_N:P_, MBN: MBP ratio.

At the 0–10 cm surface soils, the RFA showed that MB_C:P_ was the strongest factor influencing C limitation, followed by MB_N:P_ (Figure 5A). For P limitation, soil NH_4_^+^ and TN were the dominant predictors, and soil C:N also showed a significant effect (Figure 5B). At the 20–30 cm subsurface soils, soil NO_3_^−^ was the primary factor influencing C limitation, with BD as the second most important variable (Figure 5C), whereas soil MB_C:N_ was the key factor influencing P limitation, and all variables showed no significant effects (Figure 5D).

## Discussion

### Effects of tree species and soil depth on extracellular enzyme activities in subtropical plantations

Alterations in soil microbial abundance, structure, and activity can influence microbial and nutrient flows after afforestation and during aggradation (Zhou et al., 2017; Zhou et al., 2022; Lan et al., 2022; Li et al., 2024; Yang et al., 2020). Our study found that *Eucalyptus* conversion significant decreased soil C-acquiring enzyme activities compared to the control at the 0–10 cm surface soils (Figure 1A). This indicates that, compared with fast-growing *Eucalyptus* plantations, native tree species plantations reduce capacity for C decomposition, thereby suppressing microbial investment in C acquisition, potentially slowing surface-soil C turnover and increasing C stability. The observed pattern may be attributed to three main factors. First, litter from native tree species, particularly those with higher lignin contents, may be more recalcitrant than that of the previous vegetation, reducing the need for microorganisms to secrete large amounts of enzymes targeting readily decomposable C substrates. Second, shifts in tree species composition can alter the fungal-to-bacterial ratio, favoring microbial communities that are more efficient at decomposing complex carbon compounds and that therefore produce a different spectrum of extracellular enzymes. Third, the input of new litter may exert a weaker priming effect on the decomposition of pre-existing soil organic carbon, thereby lowering microbial demand for easily accessible carbon sources. Surface N-acquiring enzyme activities in *Eucalyptus* plantations were only significantly higher than those in *M. laosensis* and *M. chapensis* plantations, with no significant differences compared with the other native tree species plantations, and P-acquiring enzyme activities showed no significant differences among all plantations (Figures 1B,C). This pattern highlights that converting *Eucalyptus* to native species exerts a selective rather than uniform influence on microbial N acquisition in surface soils, with only specific species inducing detectable changes. In contrast, the stable P-acquiring enzyme activities across plantations suggest that microbial P demand and availability in surface soils are relatively conserved and less sensitive to tree species replacement.

At the 20–30 cm subsurface soils, the C- N-, and P-acquiring enzyme activities in *Eucalyptus* plantations were consistently lower than those in all native tree species plantations (Figures 1A–C), suggesting that planting native tree species systematically enhances microbial activity and nutrient cycling dynamics throughout the entire soil profile, particularly in the often-overlooked deeper soils. Unlike topsoil, which primarily depends on litter inputs, subsoil organic matter and nutrients are mainly derived from plant roots (i.e., root exudates, fine-root turnover, and rhizodeposition). Native tree species, especially deep-rooted species, can directly deliver photosynthates into deeper soil layers in the form of root exudates and dead roots, thereby providing new energy and substrates for microorganisms in deeper soils. Microbial activity is typically higher in the rhizosphere, and an increase in deep roots effectively creates more such biologically active hotspots in subsoil, collectively enhancing overall enzyme activities. In nutrient-poor subsoil, microorganisms and plant roots often form symbiotic associations (e.g., mycorrhizal fungi), jointly secreting hydrolytic enzymes to mobilize “occluded” or mineral-bound nitrogen and phosphorus. The widespread increase in enzyme activities therefore indicates an enhanced capacity of the entire system to exploit deep-soil nutrient pools. In addition, root growth can improve subsoil aeration and structure and promote water movement, thereby creating a more favorable physicochemical environment for microbial survival and activity. Collectively, restoration with native tree species enhances microbial activity and enzyme-mediated nutrient cycling throughout the soil profile, whereas *Eucalyptus* plantations exhibit vertically imbalanced nutrient cycling, which may lead to surface nutrient enrichment and subsurface depletion.

### Environmental controls on soil extracellular enzyme activities in subtropical plantations

Soil microorganism obtain essential resources by secreting extracellular enzymes, and this process is highly dependent on the nutrient availability in the environment (Wang et al., 2023; Daunoras et al., 2024; Pang et al., 2025; Yu et al., 2025). When nutrients are limited, microorganisms can secrete enzyme into the environment to mobilize the corresponding nutrients; therefore, soil nutrient status plays a pivotal role in regulating extracellular enzyme activity (Sinsabaugh et al., 2008; Kivlin and Treseder, 2014). Significant positive correlations between soil moisture and C-acquiring enzyme activity have been reported during vegetation succession in a semiarid region (Cui et al., 2020) and along afforestation chronosequences in desertified ecosystems (Bi et al., 2022). Our results showed that soil moisture exhibited a non-significant correlation with C-, N- and P-acquiring enzymes at both surface and subsurface layers (Figures 3, 4). Previous studies showed soil pH plays a key role in regulating microbial metabolism and enzyme activities, with different enzymes exhibiting distinct optimal pH ranges (Xu et al., 2017; Cui et al., 2018). Our results showed that soil pH was significantly negatively correlated with C- and P-acquiring enzyme activities (Figure 3). Acidic conditions may enhance microbial C- and P-acquisition, whereas higher soil pH may suppress related-enzyme activities in subtropical plantations, thus influencing nutrient cycling process.

Meanwhile, based on global-scale analyses, Sinsabaugh et al. (2008, 2009, 2015) demonstrated that soil extracellular enzyme activities are predominantly regulated by soil C, N and P contents and stoichiometric ratios. Our results showed that SOC was significantly negatively associated with C-acquiring enzyme activities in surface soils. In subsurface soils, SOC, C:N, and C:P were significantly positively correlated with C-acquiring enzyme activities, whereas C:N additionally showed a significant positive association with N-acquiring enzyme activities (Figure 3). Moreover, soil available nutrients can affect the metabolic demands and substrate availability of microorganisms, there altering the synthesis and secretion levels of enzymes (Cong et al., 2015; Liu G. et al., 2023; Liu M. et al., 2023). Soil NH_4_^+^ was positively, and NO_3_^−^ negatively, correlated with P-acquiring enzyme activities in two soil layers (Figure 3), the RDA results showed that NH_4_^+^ significantly affected three extracellular enzyme activities at the 20–30 cm subsurface layer (Figure 4D). Soil NH_4_^+^ can enhance microbial P demand and stimulate enzyme synthesis, thereby increasing P-acquiring enzyme activity; whereas soil NO_3_^−^ may inhibit microbial metabolic processes related to P acquisition, leading to a decrease in enzyme activity.

According to the stoichiometric balance (Elser et al., 2000), microbial biomass stoichiometry can reflect microbial nutrient deficiency (Mooshammer et al., 2014; Pang et al., 2025). Our results indicated that soil MBC, MBN and MBP were significantly and positively associated with P-acquiring enzyme activities at 0–10 cm surface soils. Soil MB_C:N_ was significantly and positively P-acquiring enzyme activities at 20–30 cm subsurface soils. The RDA results showed that soil MBC and MBN were significant and key factors influencing soil C-, N- and P-acquiring enzyme activities. Together, our results underscore that microbial biomass and stoichiometry are key determinants of soil enzyme activity in China’s subtropical plantations.

### Shifts and drivers of microbial metabolic limitation across tree species and soil depths in subtropical plantations

Tree species composition and litter quality substantially influence soil enzyme activities and microbial community structure (Liu et al., 2023; Błońska et al., 2025), thereby exerting an indirect influence on microbial resource limitation. Distinct decomposition dynamics and utilization efficiencies of cellulose, lignin, and other recalcitrant organic compounds among tree species generate considerable variability in SOC, which consequently governs the C supply available for microbial metabolism and shapes soil biochemical functioning (Owen et al., 2007; Zhou et al., 2023; Wu et al., 2023; Jing et al., 2023). C limitation is a relative concept, closely linked to the quantities of other elements (Pang et al., 2025). However, C limitation has been rarely discussed in tropical plantation forests. We observed a significant negative relationship between C-acquiring enzymes and relative C-limitation, indicating that when C supply is sufficient, microorganisms may reduce their investment in C-acquiring enzymes. Pearson correlation in surface soils (*R*^2^ = 0.73, *p* < 0.001) was significantly higher than that in subsurface soils (*R*^2^ = 0.26, *p* < 0.05), indicating that the coupling between microbial enzyme investment and C nutritional status is much stronger in surface soils than in subsurface soils. Meanwhile, we found that microbial C limitation in *Eucalyptus* plantations was only significantly higher than that in *C. hystrix* plantations in surface soils, and only higher than that in *R. championii* and *M. chapensis* plantations in subsurface soils (Figure 2D), indicating that after transformation, some native tree species indeed significantly alleviated microbial C limitation. Overall, the transformation from *Eucalyptus* to native tree species plantations optimized C supply and reshaped microbial strategies for C acquisition, thereby alleviating soil microbial C limitation to some extent.

In this study, P-limitation (vector angle >45°) was observed in soils following afforestation after clear-cutting of *Eucalyptus* plantations at both the 0–10 cm surface and 20–30 cm subsurface layers (Figure 2), consistent with the general understanding that tropical forest ecosystems are typically P-limited (Turner et al., 2018; Vallicrosa et al., 2023; Gargallo-Garriga et al., 2024). Our findings suggest that microbial P limitation is pervasive in soil microbial metabolism within tropical-subtropical plantation ecosystems, which is probably attributable to the inherently low P content in these soil regions. Furthermore, native tree species plantations exhibited greater P limitation than *Eucalyptus* plantations across both surface and subsurface layers (Figure 2C), suggesting that soils under native tree species plantations were strongly limited by P relative to those under *Eucalyptus* plantations. The main reasons are that native tree species generally have lower P contents and the soil under these species tend to have lower available P. In addition, native trees often have strong P uptake capacity, which may intensify competition with microorganisms for limited P resources, thereby imposing stronger P limitation on microbes. Moreover, native species typically produce litter more slowly and with higher lignin and cellulose contents, which decompose slowly and release P at lower rates.

The extracellular enzymatic stoichiometry model revealed that microbial metabolism in all tree species plantations underwent relative C and P limitations in soil after clear-cutting in *Eucalyptus* plantations. Furthermore, the non-significant correlation between microbial C and P limitations (Figure 2B) suggest that these limitations are not dependent in subtropical plantation forest ecosystems. Previous studies have showed that resource availability is likely a fundamental driver of microbial processes (Cherif and Loreau, 2007; She et al., 2018; Cui et al., 2021, 2024). Soil nutrient and biomass stoichiometry influences soil nutrient supply balance and microbial community structure, thereby regulating microbial metabolic processes and determining microbial metabolic limitation and its variation (Griffiths et al., 2012; Ollinger, 2011). Random forest analysis showed soil MB_C:P_ and MB_N:P_ had a significant positive correlation with C limitation at 0–10 cm surface soils, and NO_3_^−^ and BD were the dominant factors influencing C limitation at 20–30 cm subsurface soils (Figures 5A,B). Meanwhile, soil NH_4_^+^ and TN were the strongest predictor of P limitation in surface soils, whereas none of the variables showed significant effects in subsurface soils (Figures 5C,D). Our findings demonstrate that microbial C and P limitations are primarily governed by microbial biomass stoichiometric ratios and nutrient availability. In addition, higher BD might be linked to lower organic C availability or reduced microbial C acquisition, possibly leading to increased microbial C limitation. Furthermore, our analyses did not identify soil water and pH as major correlates of microbial C and P limitations in subtropical plantations (Figures 3, 5). This suggests that, under the environmental conditions of the study area, variations in soil moisture and pH were relatively small or within an optimal range, and thus their regulatory effects on microbial metabolic limitation were not pronounced.

## Conclusions and implication

Through a systematic study on the changes in forest soil enzyme activities under the transformation using native tree species, this paper draws the following key conclusions: (1) The responses of soil C-, N-, and P-acquiring enzyme activities to the transformation exhibit distinct vertical differentiation. In surface soils, C-acquiring enzyme activity generally decreased after transformation, while changes in N- and P-acquiring enzyme activities did not show significant differences in most cases, indicating that tree species transformation more strongly affects surface C cycling, while N and P cycling processes remain relatively stable. In contrast, in subsurface soils, planting different native tree species consistently and significantly enhanced all of enzymes activities, highlighting the key role of native tree species transformation in promoting nutrient mobilization and cycling in subsurface soils. (2) the patterns of soil microbial nutrient limitation undergo significant restructuring. The transformation using native tree species effectively alleviated microbial C limitation in the soil, particularly in the surface layer. However, microbial P limitation intensified in both the surface and subsurface soils. This suggests that while the transformation alleviates one limiting factor (C), it may increase the relative importance of another (P), revealing the dynamic and complex nature of ecosystem nutrient balance.

Collectively, this study demonstrates that forest transformation using native tree species can profoundly influence soil biogeochemical processes, with effects dependent on specific tree species and soil depth. Successful ecological restoration is not merely the recovery of vegetation cover, but also a process of rebalancing and efficiently reactivating the complex internal nutrient cycling engine of soils. These findings emphasize the importance of selectively adopting mixed-species plantations and paying attention to deep soil processes in ecological restoration practices to optimize soil nutrient cycling and achieve sustainable forest ecosystem recovery and management. Future research should focus on the long-term dynamics of such nutrient limitation patterns to inform nutrient balance-based forest management strategies.

## Data Availability

The raw data supporting the conclusions of this article will be made available by the authors, without undue reservation.

## References

[ref1] Bi B. Wang Y. Wang K. Zhang H. Fei H. Pan R. . (2022). Changes in microbial metabolic C–and N–limitations in the rhizosphere and bulk soils along afforestation chronosequence in desertified ecosystems. J. Environ. Manag. 303:114215. doi: 10.1016/j.jenvman.2021.114215 34864590

[ref2] Bing H. Wu Y. Zhou J. Sun H. Luo J. Wang J. . (2016). Stoichiometric variation of carbon, nitrogen, and phosphorus in soils and its implication for nutrient limitation in alpine ecosystem of eastern Tibetan plateau. J. Soils Sediments 16, 405–416. doi: 10.1007/s11368-015-1200-9

[ref3] Błońska E. Lasota J. Prażuch W. Ilek A. (2025). Vertical variations in enzymatic activity and C:N:P stoichiometry in forest soils under the influence of different tree species. Eur. J. For. Res. 144, 83–94.

[ref4] Bowles T. M. Acosta–Martinez V. Calderon F. Jackson L. E. (2014). Soil enzyme activities, microbial communities, and carbon and nitrogen availability in organic agroecosystems across an intensively–managed agricultural landscape. Soil Biol. Biochem. 68, 252–262. doi: 10.1016/j.soilbio.2013.10.004

[ref5] Breiman L. Cutler A. Liaw A. Wiener M. (2018). Package “randomforest”, vol. 81. Berkeley, CA: University of California, Berkeley, 1–29.

[ref6] Brookes P. C. (1995). The use of microbial parameters in monitoring soil pollution by heavy metals. Biol. Fertil. Soils 19, 269–279. doi: 10.1007/bf00336094

[ref7] Cherif M. Loreau M. (2007). Stoichiometric constraints on resource use, competitive interactions, and elemental cycling in microbial decomposers. Am. Nat. 169, 709–724. doi: 10.1086/516844, 17479458

[ref8] Cong J. Liu X. Lu H. Xu H. Li Y. Deng Y. . (2015). Available nitrogen is the key factor influencing soil microbial functional gene diversity in tropical rainforest. BMC Microbiol. 15:167. doi: 10.1186/s12866-015-0491-8, 26289044 PMC4546036

[ref9] Cui Y. Bing H. Fang L. Jiang M. Shen G. Yu J. . (2021). Extracellular enzyme stoichiometry reveals the carbon and phosphorus limitations of microbial metabolisms in the rhizosphere and bulk soils in alpine ecosystems. Plant Soil 458, 7–20. doi: 10.1007/s11104-019-04159-x

[ref10] Cui Y. Bing H. Moorhead D. L. Delgado-Baquerizo M. Ye L. Yu J. . (2022). Ecoenzymatic stoichiometry reveals widespread soil phosphorus limitation to microbial metabolism across Chinese forests. Commun. Earth Environ. 3:184. doi: 10.1038/s43247-022-00523-5

[ref11] Cui Y. Fang L. Guo X. Wang X. Zhang Y. Li P. . (2018). Ecoenzymatic stoichiometry and microbial nutrient limitation in rhizosphere soil in the arid area of the northern loess plateau, China. Soil Biol. Biochem. 116, 11–21. doi: 10.1016/j.soilbio.2017.09.025

[ref2001] Cui Y. Zhang Y. Duan C. Wang X. Zhang X. Ju W. . (2020). Ecoenzymatic stoichiometry reveals microbial phosphorus limitation decreases the nitrogen cycling potential of soils in semi-arid agricultural ecosystems. Soil Till. Res. 197:104463. doi: 10.1016/j.still.2019.104463

[ref12] Cui Y. Hu J. Peng S. Delgado-Baquerizo M. Moorhead D. L. Sinsabaugh R. L. . (2024). Limiting resources define the global pattern of soil microbial carbon use efficiency. Adv. Sci. 11:2308176. doi: 10.1002/advs.202308176 PMC11425281 39024521

[ref13] Daunoras J. Kačergius A. Gudiukaitė R. (2024). Role of soil microbiota enzymes in soil health and activity changes depending on climate change and the type of soil ecosystem. Biology 13:85. doi: 10.3390/biology13020085, 38392304 PMC10886310

[ref14] Deng Q. Cheng X. Hui D. Zhang Q. Li M. Zhang Q. (2016). Soil microbial community and its interaction with soil carbon and nitrogen dynamics following afforestation in Central China. Sci. Total Environ. 541, 230–237. doi: 10.1016/j.scitotenv.2015.09.080, 26410698

[ref15] Deng L. Peng C. Huang C. Wang K. Liu Q. Liu Y. . (2019). Drivers of soil microbial metabolic limitation changes along a vegetation restoration gradient on the loess plateau, China. Geoderma 353, 188–200.

[ref16] Elser J. J. Sterner R. W. Gorokhova E. Fagan W. F. Markow T. A. Cotner J. B. . (2000). Biological stoichiometry from genes to ecosystems. Ecol. Lett. 3, 540–550. doi: 10.1111/j.1461-0248.2000.00185.x

[ref17] Gargallo-Garriga A. Sardans J. Llusià J. Peguero G. Ayala-Roque M. Courtois E. A. . (2024). Different profiles of soil phosphorous compounds depending on tree species and availability of soil phosphorus in a tropical rainforest in French Guiana. BMC Plant Biol. 24:278. doi: 10.1186/s12870-024-04907-x 38609866 PMC11010349

[ref18] Geisseler D. Horwath W. R. Scow K. M. (2011). Soil moisture and plant residue addition interact in their effect on extracellular enzyme activity. Pedobiologia 54, 71–78. doi: 10.1016/j.pedobi.2010.10.001

[ref19] Griffiths B. S. Spilles A. Bonkowski M. (2012). C:N:P stoichiometry and nutrient limitation of the soil microbial biomass in a grazed grassland site under experimental P limitation or excess. Ecol. Process. 1:6. doi: 10.1186/2192-1709-1-6

[ref20] Jing Y. Zhao X. Liu S. Tian P. Sun Z. Chen L. . (2023). Influence of tree species on soil microbial residue accumulation and distribution among soil aggregates in subtropical plantations of China. Ecol. Process. 12:32. doi: 10.1186/s13717-023-00444-x

[ref21] Kivlin S. N. Treseder K. K. (2014). Soil extracellular enzyme activities correspond with abiotic factors more than fungal community composition. Biogeochemistry 117, 23–37. doi: 10.1007/s10533-013-9852-2

[ref22] Lan J. Wang S. Wang J. Qi X. Long Q. Huang M. (2022). The shift of soil bacterial community after afforestation influence soil organic carbon and aggregate stability in karst region. Front. Microbiol. 13:901126. doi: 10.3389/fmicb.2022.901126, 35832811 PMC9271926

[ref23] Li L. Wang D. Liu X. Zhang B. Liu Y. Xie T. . (2014). Soil organic carbon fractions and microbial community and functions under changes in vegetation: a case of vegetation succession in karst forest. Environ. Earth Sci. 71, 3727–3735. doi: 10.1007/s12665-013-2767-3

[ref24] Li Y. Wang B. Zhang Y. Ao D. Feng C. Wang P. . (2024). Afforestation increased the microbial necromass carbon accumulation in deep soil on the loess plateau. J. Environ. Manag. 349:119508. doi: 10.1016/j.jenvman.2023.119508, 37952382

[ref25] Liang C. Schimel J. P. Jastrow J. D. (2017). The importance of anabolism in microbial control over soil carbon storage. Nat. Microbiol. 2, 1–6. doi: 10.1038/nmicrobiol.2017.105, 28741607

[ref26] Liu M. Huang F. Huang Y. Gan X. Li Y. Wang M. (2023). Soil nutrient availability regulates microbial community composition and enzymatic activities at different soil depths along an elevation gradient in the Nanling nature reserve, China. Forests 14:1514. doi: 10.3390/f14081514

[ref27] Liu G. Wang H. Yan G. Wang M. Jiang S. Wang X. . (2023). Soil enzyme activities and microbial nutrient limitation during the secondary succession of boreal forests. Catena 230:107268. doi: 10.1016/j.catena.2023.107268

[ref28] Malik A. A. Martiny J. B. H. Brodie E. L. Martiny A. C. Treseder K. K. Allison S. D. (2020). Defining trait–based microbial strategies with consequences for soil carbon cycling under climate change. ISME J. 14, 1–9. doi: 10.1038/s41396-019-0510-0, 31554911 PMC6908601

[ref29] Moorhead D. L. Rinkes Z. L. Sinsabaugh R. L. Weintraub M. N. (2013). Dynamic relationships between microbial biomass, respiration, inorganic nutrients and enzyme activities: informing enzyme-based decomposition models. Front. Microbiol. 4:223. doi: 10.3389/fmicb.2013.00223 23964272 PMC3740267

[ref30] Moorhead D. L. Sinsabaugh R. L. Hill B. H. Weintraub M. N. (2016). Vector analysis of ecoenzyme activities reveal constraints on coupled C, N and P dynamics. Soil Biol. Biochem. 93, 1–7. doi: 10.1016/j.soilbio.2015.10.019

[ref31] Mooshammer M. Wanek W. Zechmeister-Boltenstern S. Richter A. (2014). Stoichiometric imbalances between terrestrial decomposer communities and their resources: mechanisms and implications of microbial adaptations to their resources. Front. Microbiol. 5:22. doi: 10.3389/fmicb.2014.00022, 24550895 PMC3910245

[ref32] Naveed M. Herath L. Moldrup P. Arthur E. Nicolaisen M. Norgaard T. . (2016). Spatial variability of microbial richness and diversity and relationships with soil organic carbon, texture and structure across an agricultural field. Appl. Soil Ecol. 103, 44–55. doi: 10.1016/j.apsoil.2016.03.004

[ref33] Ollinger S. V. (2011). Sources of variability in canopy reflectance and the convergent properties of plants. New Phytol. 189, 375–394. doi: 10.1111/j.1469-8137.2010.03536.x 21083563

[ref34] Owen K. E. Tenhunen J. Reichstein M. Wang Q. Falge E. Geyer R. . (2007). Linking flux network measurements to continental scale simulations: ecosystem carbon dioxide exchange capacity under non-water-stressed conditions. Glob. Change Biol. 13, 734–760. doi: 10.1111/j.1365-2486.2007.01326.x

[ref35] Pang C. Zhang Z. Zhu X. Wei W. Mustafa A. Chen W. . (2025). Divergent microbial metabolic limitations across soil depths after two decades of high nitrogen inputs in a primary tropical forest. Glob. Change Biol. 31:e70440. doi: 10.1111/gcb.70440 40827725

[ref36] Peng X. Wang W. (2016). Stoichiometry of soil extracellular enzyme activity along a climatic transect in temperate grasslands of northern China. Soil Biol. Biochem. 98, 74–84. doi: 10.1016/j.soilbio.2016.04.008

[ref37] Prescott C. E. (2010). Litter decomposition: what controls it and how can we alter it to sequester more carbon in forest soils? Biogeochemistry 101, 133–149. doi: 10.1007/s10533-010-9439-0

[ref38] Prescott C. E. Grayston S. J. (2013). Tree species influence on microbial communities in litter and soil: current knowledge and research needs. Soil Biol. Biochem. 57, 349–357. doi: 10.1016/j.foreco.2013.02.034

[ref39] R Core Team (2019) R: A language and environment for statistical computing (v. 3.5.3). R foundation for statistical computing, Vienna, Austria. Available online at: https://www.R-project.org/ (Accessed March 11, 2019).

[ref40] Rosinger C. Rousk J. Sandén H. (2019). Can enzymatic stoichiometry be used to determine growth–limiting nutrients for microorganisms?–a critical assessment in two subtropical soils. Soil Biol. Biochem. 128, 115–126. doi: 10.1016/j.soilbio.2018.10.011

[ref41] Saiya–Cork K. R. Sinsabaugh R. L. Zak D. R. (2002). The effects of long term nitrogen deposition on extracellular enzyme activity in an *Acer saccharum* forest soil. Soil Biol. Biochem. 34, 1309–1315. doi: 10.1016/S0038-0717(02)00074-3

[ref42] Schaap K. J. Fuchslueger L. Quesada C. A. Hofhansl F. Valverde B. O. Camargo P. R. . (2023). Seasonal fluctuations of extracellular enzyme activities are related to the biogeochemical cycling of C, N and P in a tropical terra–firme forest. Biogeochemistry 163, 1–15. doi: 10.1007/s10533-022-01009-4

[ref43] She W. Bai Y. Zhang Y. Qin S. Feng W. Sun Y. . (2018). Resource availability drives responses of soil microbial communities to short–term precipitation and nitrogen addition in a desert shrubland. Front. Microbiol. 9:186. doi: 10.3389/fmicb.2018.00186, 29479346 PMC5811472

[ref44] Sinsabaugh R. L. Hill B. H. Follstad Shah J. J. (2009). Ecoenzymatic stoichiometry of microbial organic nutrient acquisition in soil and sediment. Nature 462, 795–798. doi: 10.1038/nature08632 20010687

[ref45] Sinsabaugh R. L. Lauber C. L. Weintraub M. N. Ahmed B. Allison S. D. Crenshaw C. . (2008). Stoichiometry of soil enzyme activity at global scale. Ecol. Lett. 11, 1252–1264. doi: 10.1111/j.1461-0248.2008.01245.x, 18823393

[ref46] Sinsabaugh R. L. Shah J. J. F. (2012). Ecoenzymatic stoichiometry and ecological theory. Annu. Rev. Ecol. Evol. Syst. 43, 313–343. doi: 10.1146/annurev-ecolsys-071112-124414

[ref47] Sinsabaugh R. L. Shah J. J. F. Findlay S. G. Kuehn K. A. Moorhead D. L. (2015). Scaling microbial biomass, metabolism and resource supply. Biogeochemistry 122, 175–190. doi: 10.1007/s10533-014-0058-z

[ref48] Šmilauer P. Lepš J. (2014). Multivariate analysis of ecological data using CANOCO 5. Cambridege, UK: Cambridge University Press.

[ref49] Snajdr J. Valaskova V. Merhautova V. Herinkova J. Cajthaml T. Baldrian P. (2008). Spatial variability of enzyme activities and microbial biomass in the upper layers of *Quercus petraea* forest soil. Soil Biol. Biochem. 40, 2068–2075. doi: 10.1016/j.soilbio.2008.01.015

[ref50] Sun H. Sun F. Deng X. Naleen S. (2025). Soil carbon fractions drive microbial community assembly processes during forest succession. J. Environ. Manag. 373:123638. doi: 10.1016/j.jenvman.2024.123638 39667340

[ref51] Sveen T. R. Viketoft M. Bengtsson J. Strengbom J. Lejoly J. Buegger F. . (2025). Functional diversity of soil microbial communities increases with ecosystem development. Nat. Commun. 10:10408. doi: 10.1038/s41467-025-66544-8 PMC12644890 41274910

[ref52] Tapia–Torres Y. Elser J. J. Souza V. García–Oliva F. (2015). Ecoenzymatic stoichiometry at the extremes: how microbes cope in an ultra–oligotrophic desert soil. Soil Biol. Biochem. 87, 34–42. doi: 10.1016/j.soilbio.2015.04.007

[ref53] Tian J. McCormack L. Wang J. Guo D. Wang Q. Zhang X. . (2015). Linkages between the soil organic matter fractions and the microbial metabolic functional diversity within a broad-leaved Korean pine forest. Eur. J. Soil Biol. 66, 57–64. doi: 10.1016/j.ejsobi.2014.12.001

[ref54] Turner B. L. Brenes–Arguedas T. Condit R. (2018). Pervasive phosphorus limitation of tree species but not communities in tropical forests. Nature 555, 367–370. doi: 10.1038/nature25789 29513656

[ref55] Ushio M. Wagai R. Balser T. C. Kitayama K. (2010). Variations in the soil microbial community composition of a tropical montane forest ecosystem: Does tree species matter? Soil Biol. Biochem. 40, 2699–2702. doi: 10.1016/j.soilbio.2008.06.023

[ref56] Vallicrosa H. Lugli L. F. Fuchslueger L. Sardans J. RamirezRojas L. Verbruggen E. . (2023). Phosphorus scarcity contributes to nitrogen limitation in lowland tropical rainforests. Ecology 104:e4049. doi: 10.1002/ecy.4049 37039427

[ref57] Wang X. Li Y. Wang L. Duan Y. Yao B. Chen Y. . (2023). Soil extracellular enzyme stoichiometry reflects microbial metabolic limitations in different desert types of northwestern China. Sci. Total Environ. 874:162504. doi: 10.1016/j.scitotenv.2023.162504, 36863586

[ref58] Waring B. S. Weintraub R. Sinsabaugh (2013). Ecoenzymatic stoichiometry of microbial nutrient acquisition in tropical soils. Biogeochemistry 117, 101–113. doi: 10.1007/s10533-013-9849-x

[ref59] Wei T. Simko V. Levy M. Xie Y. Jin Y. Zemla J. (2017). Package “corrplot”. Stat 56:e24.

[ref60] Wu D. Yin C. Fan Y. Chi H. Liu Z. Jin G. (2023). Effect of forest planting patterns on the formation of soil organic carbon during litter lignocellulose degradation from a microbial perspective. Front. Microbiol. 14:1327481. doi: 10.3389/fmicb.2023.1327481, 38188580 PMC10771852

[ref61] Xu M. Li X. Kuyper T. W. Xu M. Zhang J. (2021). High microbial diversity stabilizes the responses of soil organic carbon decomposition to warming in the subsoil on the Tibetan plateau. Glob. Chang. Biol. 27, 2061–2075. doi: 10.1111/gcb.15553, 33560552

[ref62] Xu M. Wang G. Li X. Cai X. Li X. Christie P. . (2015). The key factor limiting plant growth in cold and humid alpine areas also plays a dominant role in plant carbon isotope discrimination. Front. Plant Sci. 3:961. doi: 10.3389/fpls.2015.00961 PMC4630956 26579188

[ref63] Xu Z. Yu G. Zhang X. He N. Wang Q. Wang S. . (2017). Soil enzyme activity and stoichiometry in forest ecosystems along the north–south transect in eastern China (NSTEC). Soil Biol. Biochem. 104, 152–163. doi: 10.1016/j.soilbio.2016.10.020

[ref64] Yang Y. Liang C. Wang Y. Cheng H. An S. Chang S. X. (2020). Soil extracellular enzyme stoichiometry reflects the shift from P–to N–limitation of microorganisms with grassland restoration. Soil Biol. Biochem. 149:107928. doi: 10.1016/j.soilbio.2020.107928

[ref65] Yu J. Shi P. Zong N. Cui Y. Hou G. Chen X. . (2025). The desertification process alters soil microbial metabolic limitations and their effects on soil carbon sequestration in a Tibetan alpine steppe. J. Integr. Agric. 24, 845–858. doi: 10.1016/j.jia.2024.07.038

[ref66] Zhang Y. Cui D. Yang H. Nijat K. (2020). Differences of soil enzyme activities and its influencing factors under different flooding conditions in Ili Valley, Xinjiang. PeerJ 8:e8531. doi: 10.7717/peerj.8531 32201637 PMC7073240

[ref67] Zhang W. Liu W. Xu M. Deng J. Han X. Yang G. . (2019a). Response of forest growth to C:N:P stoichiometry in plants and soils during *Robinia pseudoacacia* afforestation on the loess plateau, China. Geoderma 337, 280–289. doi: 10.1016/j.geoderma.2018.09.042

[ref68] Zhang W. Xu Y. Gao D. Wang X. Liu W. Deng J. . (2019b). Ecoenzymatic stoichiometry and nutrient dynamics along a revegetation chronosequence in the soils of abandoned land and *Robinia pseudoacacia* plantation on the loess plateau, China. Soil Biol. Biochem. 134, 1–14. doi: 10.1016/j.soilbio.2019.03.017

[ref69] Zhao F. Z. Ren C. J. Han X. H. Yang G. H. Wang J. Doughty R. (2018). Changes of soil microbial and enzyme activities are linked to soil C, N and P stoichiometry in afforested ecosystems. For. Ecol. Manag. 427, 289–295. doi: 10.1016/j.foreco.2018.06.011

[ref70] Zhou G. Wan J. Gu Z. Ding W. Hu S. Du Q. . (2023). Functional diversity accelerates the decomposition of litter recalcitrant carbon but reduces the decomposition of labile carbon in subtropical forests. Forests 14:2258. doi: 10.3390/f14112258

[ref71] Zhou X. Wang S. S. J. Chen C. (2017). Modeling the effects of tree species and incubation temperature on soil's extracellular enzyme activity in 78–year–old tree plantations. Biogeosciences 14, 5393–5402. doi: 10.5194/bg-14-5393-2017

[ref72] Zhou S. Wang J. Chen L. Wang J. Zhao F. (2022). Microbial community structure and functional genes drive soil priming effect following afforestation. Sci. Total Environ. 825:153925. doi: 10.1016/j.scitotenv.2022.153925 35218819

