# Supplementary material for: Effects of converting Eucalyptus plantations to six native tree species on microbial nutrient limitation in subtropical plantation soils
Source: Front Microbiol. 2026 Mar 16;17:1770355. doi: 10.3389/fmicb.2026.1770355 (PMC13033790; doi:10.3389/fmicb.2026.1770355)
Supplement: Supplementary file 1 [file Table_1.docx]

# Supplementary Material

# Effects of converting *Eucalyptus* plantations to six native tree species on microbial nutrient limitation in subtropical plantation soils

# Yongmei Xiong^1,2,3,4^, Seping Dai^3,4^, Yu Su^3,4^, Yanqiong Li^3,4^, and Jianmin Xu^1*^

1 Research Institute of Tropical Forestry, Chinese Academy of Forestry, Guangzhou, China, 2 Nanjing Forestry University, Nanjing, China. 3 Guangzhou Institute of Forestry and Landscape Architecture, Guangzhou, China. 4 Guangzhou Collaborative Innovation Center on Science-Tech of Ecology and Landscape, Guangzhou, China

Table 1 Soil properties of 0-10cm surface soils under different tree species plantations.

| Sol | ER | MM | RC | EF | ML | CH | MC |
| --- | --- | --- | --- | --- | --- | --- | --- |
| pH | 4.70±0.10a | 4.40±0.10b | 4.83±0.32a | 4.77±0.15a | 4.73±0.15a | 4.83±0.12a | 4.57±0.06ab |
| BD | 1.03±0.10a | 1.13±0.05a | 1.05±0.09a | 1.0±0.12a | 1.10±0.09a | 0.99±0.08a | 1.16±0.11a |
| SWC | 13.21±0.98ab | 16.17±2.84a | 17.27±1.61a | 15.78±0.68ab | 16.44±1.48a | 16.97±3.70a | 12.01±2.11b |
| C | 15.78±2.34bc | 20.59±1.47a | 14.03±2.11cd | 16.63±2.54bc | 10.86±1.46d | 18.53±2.37ab | 16.89±1.35bc |
| N | 0.96±0.24bc | 1.30±0.48ab | 0.78±0.31bc | 0.42±0.02c | 0.70±0.32bc | 1.33±0.60ab | 1.71±0.22a |
| P | 0.26±0.05b | 0.32±0.05ab | 0.34±0.08ab | 0.31±0.03ab | 0.40±0.06a | 0.38±0.08ab | 0.34±0.11ab |
| C:N | 16.82±2.32b | 17.37±6.04b | 19.54±6.27b | 39.53±7.72a | 18.33±9.27b | 16.37±8.33b | 9.94±0.51b |
| C:P | 61.76±16.68a | 65.16±14.26a | 42.18±6.86ab | 53.09±6.45a | 27.47±4.95b | 50.37±10.47ab | 54.14±20.37a |
| N:P | 3.80±1.42ab | 3.94±0.88ab | 2.26±0.47bc | 1.36±0.21c | 1.83±0.97bc | 3.48±1.40abc | 5.50±2.28a |
| NH4+ | 12.97±3.33abc | 9.76±4.44bc | 9.30±2.95c | 15.31±1.10ab | 8.08±1.99c | 17.59±3.40a | 13.52±2.90abc |
| NO3− | 1.00±0.05cd | 12.01±3.01a | 7.84±4.33b | 1.14±0.84cd | 5.97±1.74b | 0.51±0.49d | 4.91±1.44bc |
| AVP | 1.50±1.47a | 2.03±0.92a | 3.17±2.25a | 2.63±2.02a | 1.27±2.02a | 1.03±1.27a | 0.53±0.59a |
| MBC | 169.93±26.38a | 165.16±14.86a | 63.98±17.09b | 176.80±60.06a | 90.58±68.20b | 188.84±23.73a | 51.61±25.82b |
| MBN | 17.58±5.38ab | 17.95±4.11ab | 3.70±1.04c | 24.14±11.69a | 12.32±1.67bc | 24.50±2.32a | 4.76±2.26c |
| MBP | 4.52±2.04a | 3.13±0.84abc | 0.97±0.11c | 3.67±0.89ab | 2.87±0.31abc | 5.10±2.19a | 1.31±0.69bc |
| MB_C:N_ | 10.05±2.08b | 9.44±1.58b | 17.52±3.40a | 7.82±1.58b | 7.20±5.26b | 7.69±0.24b | 10.69±1.21b |
| MB_C:P_ | 43.36±20.28a | 56.43±21.11a | 66.45±19.08a | 47.68±10.83a | 31.17±23.24a | 42.59±19.78a | 40.01±4.06a |
| MB_N:P_ | 4.18±1.04a | 6.19±2.89a | 3.79±0.75a | 6.32±1.98a | 4.29±0.13a | 5.56±2.68a | 3.76±0.35a |

*ER, Eucalyptus robusta*; *MM*, *Michelia macclurei*; RC, *Rhodoleia championii*, EF, *Erythrophleum fordii*, ML, *Mytilaria laosensis*; CH, *Castanopsis hystrix*; MC, *Michelia chapensis*; BD, bulk density; SWC, soil water content; SOC, soil organic carbon; TN, total nitrogen; TP, total phosphorus; C:N, SOC: TN ratio; C:P, SOC: TP ratio; N:P, TN: TP ratio; AVP, available phosphorus; NH_4_^+^; ammonium nitrogen; NO_3_^−^, nitrate nitrogen; MBC, microbial biomass carbon; MBN, microbial biomass nitrogen; MBP, microbial biomass phosphorus; MB_C:N_, MBC: MBN ratio; MB_C:P_, MBC: MBP ratio; MB_N:P_, MBN: MBP ratio.

Table 2 Soil properties of 20-30cm deeper soils under different tree species plantations.

| Soil | ER | MM | RC | EF | ML | CH | MC |
| --- | --- | --- | --- | --- | --- | --- | --- |
| pH | 4.73±0.15ab | 4.60±0.13b | 4.80±0.30ab | 4.77±0.15ab | 4.90±0.10a | 4.73±0.06ab | 4.67±0.06ab |
| BD | 1.29±0.07ab | 1.11±0.05b | 1.26±0.11ab | 1.30±0.07ab | 1.33±0.15a | 1.28±0.05ab | 1.20±0.16ab |
| SWC | 11.98±1.19c | 15.30±2.83ab | 14.30±1.04abc | 12.67±0.66bc | 16.41±0.44a | 13.60±1.41abc | 11.58±1.59c |
| C | 6.59±2.22ab | 9.55±2.58a | 6.28±0.79ab | 5.74±1.55bc | 2.64±0.55c | 5.94±0.96b | 7.52±2.60ab |
| N | 0.63±0.25ab | 0.56±0.11ab | 0.82±0.29a | 0.26±0.06b | 0.46±0.04ab | 0.57±0.27ab | 0.80±0.39a |
| P | 0.25±0.04b | 0.34±0.04ab | 0.38±0.06a | 0.28±0.06ab | 0.30±0.06ab | 0.34±0.09ab | 0.31±0.03ab |
| C:N | 10.61±1.15c | 17.11±2.46ab | 8.26±2.62c | 21.61±2.09a | 5.74±0.72c | 11.49±3.91bc | 10.96±6.19c |
| C:P | 27.10±9.71a | 28.15±5.16a | 17.09±5.18ab | 21.76±8.96ab | 9.27±3.24b | 18.27±4.99ab | 24.32±6.87a |
| N:P | 2.59±1.04a | 1.66±0.29a | 2.25±1.01a | 0.99±0.35a | 1.59±0.40a | 1.88±1.30a | 2.53±1.02a |
| NH4^+^ | 5.06±1.46a | 4.43±1.56a | 4.93±1.62a | 4.90±1.52a | 3.54±1.19a | 4.74±0.93a | 5.33±1.30a |
| NO3− | 0.19±0.16c | 2.92±1.36a | 3.14±0.21a | 0.93±0.12bc | 2.37±0.94ab | 1.16±0.23bc | 2.94±1.32a |
| AVP | 1.17±0.64a | 1.10±0.85a | 1.90±1.61a | 1.10±1.56a | 1.00±1.39a | 0.27±0.12a | 0.33±0.25a |
| MBC | 42.88±18.53ab | 98.03±57.95a | 32.73±14.26ab | 86.94±71.96ab | 16.99±13.83b | 60.43±21.03ab | 22.21±17.48b |
| MBN | 4.79±2.45a | 12.71±11.53a | 4.18±2.42a | 15.49±19.27a | 3.89±2.11a | 6.95±3.20a | 3.93±1.88a |
| MBP | 1.53±0.68a | 1.82±0.61a | 0.48±0.32a | 1.85±1.35a | 1.36±0.33a | 1.72±0.80a | 0.96±0.47a |
| MB_C:N_ | 9.26±1.04a | 9.21±2.80a | 9.60±5.69a | 8.39±3.84a | 5.11±3.23a | 8.96±1.12a | 5.18±2.05a |
| MB_C:P_ | 28.43±6.20b | 50.87±13.62ab | 95.69±70.25a | 44.71±8.37ab | 14.69±14.95b | 36.39±4.66b | 21.25±8.16b |
| MB_N:P_ | 3.15±1.07a | 6.22±3.74a | 15.21±16.69a | 6.42±4.05a | 2.94±1.65a | 4.06±0.02a | 4.11±0.07a |

*ER, Eucalyptus robusta*; *MM, Michelia macclurei*; RC, *Rhodoleia championii*, EF, *Erythrophleum fordii*, ML, *Mytilaria laosensis*; CH, *Castanopsis hystrix*; MC, *Michelia chapensis*; *ER, Eucalyptus robusta*; *MM*, *Michelia macclurei*; RC, *Rhodoleia championii*, EF, *Erythrophleum fordii*, ML, *Mytilaria laosensis*; CH, *Castanopsis hystrix*; MC, *Michelia chapensis*; BD, bulk density; SWC, soil water content; SOC, soil organic carbon; TN, total nitrogen; TP, total phosphorus; C:N, SOC: TN ratio; C:P, SOC: TP ratio; N:P, TN: TP ratio; AVP, available phosphorus; NH_4_^+^; ammonium nitrogen; NO_3_^−^, nitrate nitrogen; MBC, microbial biomass carbon; MBN, microbial biomass nitrogen; MBP, microbial biomass phosphorus; MB_C:N_, MBC: MBN ratio; MB_C:P_, MBC: MBP ratio; MB_N:P_, MBN: MBP ratio.
